# A focus group study for the design of a web-based tool for improving problem-solving in older adults

**DOI:** 10.1007/s10433-024-00814-0

**Published:** 2024-06-13

**Authors:** Sabrina Cipolletta, Dario Signorello, Sara Zuppiroli, Alexandra Hering, Nicola Ballhausen, Giovanna Mioni, Matthias Kliegel, Mauro Gaspari, Franca Stablum

**Affiliations:** 1 Department of General Psychology, University of Padova, Padua, Italy; 2 Department of Computer Science and Engineering, University of Bologna, Bologna, Italy; 3 Department of Developmental Psychology, Tilburg University, Tilburg, Netherlands; 4 Faculty of Psychology and Educational Sciences, University of Geneva, Geneva, Switzerland

**Keywords:** Active aging, Cognitive training, Ecological validity, eHealth, Online intervention

## Abstract

The development of easily accessible and usable social and cognitive enhancement trainings is becoming a priority to reduce the impact of aging on quality of life. Since most activities of daily living (e.g., making a meal) require problem-solving skills, problem-solving interventions could be used to improve and/or maintain functional abilities in aging to prolong independence. To design an effective problem-solving training and increase older adults' adherence to the training, this study examined older adults' perceptions of their challenges in activities of daily living, their skills and difficulties in using information technology (IT), and their motivations and expectations for participating in a web-based problem-solving training activity. Four focus groups (two in Italy and two in the Netherlands) were conducted with older adults aged between 65 and 84 years, a total of 27 participants. The data were analyzed using the Atlas.ti 8 software for the thematic analysis. The analysis identified five thematic areas: interests and activities, difficulties and concerns, experiences and motivations for training, expertise and resources, suggestions for the design of the new training. The results were used to develop a first prototype of a Shared, Web-based, Intelligent Flexible Thinking Training (SWIFT), adapted to future user needs. The participation of older adults in this design phase was critical to understanding their needs, motivations, and expectations regarding the implementation and use of a cognitive enhancement training.

## Background

The increase in the average age of the population raises important challenges for the society, such as the increasing incidence of age-related cognitive decline (GBD 2019 Dementia Forecasting Collaboratos 2022). Therefore, it has been suggested that countries experiencing a sharp increase in the average age should implement training and interventions to slow cognitive decline and allow older people to maintain some independence in their daily lives, thus promoting active aging (Depp et al. 2012). Previous studies have demonstrated the efficacy of approaches aimed at improving cognitive abilities to promote cognitive health and participation in social life among older people, thereby improving their quality of life (Wilson et al. 2012; Mewborn et al. 2017; Nguyen et al. 2022).

An important aspect of independence in old age is maintaining the ability to solve everyday problems. Any task that requires planning, organization, memorization, time management, and flexible thinking can become especially challenging for older adults. In everyday life, this means that a person's ability to care for themselves, complete tasks, keep appointments, and interact appropriately with others may be compromised.

Often, retirement and withdrawal from productive activities cause older adults to limit their activities and not use problem-solving skills as they once did. Thus, problem-solving barriers (i.e., something that prevents people from finding a successful solution to a problem) become more likely in older adulthood (Charness and Boot 2009). However, if problem solving abilities remain intact, an individual can experience significant cognitive loss and still function independently and productively (Lezak et al. 2012).

There is growing recognition of the potential benefits of information technology (IT) use in improving the health, safety, well-being, and social participation of older adults (Schaie and Charness 2003; Burdick and Sunkyo 2004; Cotten et al. 2014; Hasan and Linger 2016). However, the last two decades have seen conflicting results regarding older adults' behavior towards new technologies (Huang and Oteng 2023). Some studies have suggested that older people do not use certain technologies as much as younger people (Adler 2006; Czaja et al. 2006) and that their experiences and attitudes towards new technologies are negative, especially when compared to younger generations (Timmerman 1998). On the contrary, other studies have shown that older people are open to using IT, pointing out that their use could be hindered by some variables related to both aging (e.g., cognitive decline) and IT itself (e.g., interface usability) (Vaportzis et al. 2017; Roberts et al. 2019; Vulpe and Crăciun 2019). For example, Heinz et al. (2013) showed that older adults are not negatively biased toward IT, but rather have a desire to gather information about the potential benefits of IT in maintaining a high quality of life and preserving or even enhancing their physical and mental abilities. The authors also emphasized that older adults were willing to adopt new technologies if the benefits derived from their use outweighed the feelings of inadequacy generated by the use of such technologies. In addition, Mitzner et al. (2010) provided converging evidence that ease of use and usefulness of technology training are significant and critical factors in predicting acceptance and subsequent use of IT. Finally, we believe it is important to remember that IT use also depends on resources such as education and socioeconomic level (i.e. higher income; Huxhold et al. 2020). These digital inequalities require targeted interventions (Fang et al. 2019), while the grey digital divide requires the promotion of digital literacy for the ageing population.

In an attempt to explain the reasons behind the non-acceptance and non-use of technological tools by older people and tackle digital inequalities, it is argued that technological devices should focus more on users' characteristics, needs, and preferences (Chen and Chan 2013). A promising way to address this issue is to involve users (also those from disadvantaged groups) in the technological development process through a participatory approach (Spinuzzi 2005). A participatory approach requires strong user involvement in all stages of the research and design process (Merkel and Kucharski 2019), and participants should be given the opportunity to directly influence design decisions in the development process. In fact, the life experiences of users are considered equally relevant to the expert knowledge of designers and researchers. The main goal of involving older people in IT design is to better understand their needs, expectations, and preferences in the hope of developing technologies that better meet the needs of future users (Fischer et al. 2020). Consequently, user involvement throughout the technological design process is expected to increase the acceptance of the final product and promote its adoption (Merkel and Kucharski 2019; Rogers et al. 2022).

The aim of this study was to understand older people’s needs, possible issues and motivations for participating in a web-based cognitive training to boost the design of a new intelligent web-based training program to improve problem-solving skills in older adults (Shared, Web-based, Intelligent Flexible Thinking Training, SWIFT). This training includes cognitive exercises designed to assess and improve cognitive functions underlying problem-solving, in particular planning, with high ecological validity. Exercises with high ecological validity are exercises that train participants on typical tasks required in everyday life, with a potential positive impact on participants' quality of life (Moreau and Conway 2014). There are many activities that require planning and could be used to develop problem solving skills: shopping, meeting a friend, leisure activities in the city centre, looking after the grandchild, going to the doctor, etc. Some of these activities have been used as scenarios for the development of cognitive training exercises. For example, in the plan-a-day task (Funke and Kruger, 1995; Holt et al. 2011; Baschieri et al. 2018; Gaspari et al. 2024), the task was to plan a list of daily life tasks to be completed during a day, taking into account different constraints. Other examples are the Zoo Map visit (Gaspari et al. 2024) or a shopping exercise implemented in the Rehacom cognitive training system (López-Martínez, 2011). There is definitely an element of arbitrariness in choosing which activity to focus on, with advantages and disadvantages to each choice and a lack of systematic comparison of the effectivity of these different activities.

The activity of planning a trip is inherently more complex than the examples mentioned above, as it can span multiple days and involve the coordination of various activities (e.g., selecting a train and purchasing a ticket, organizing daily activities). This scenario was chosen because it presents a challenging yet familiar task that includes fundamental planning elements encountered in everyday life, such as travel logistics, time management, and budgeting. Simultaneously, it encourages users to explore new destinations, pushing them beyond their usual boundaries. Planning a trip not only provides an opportunity to learn about cities they may not typically visit but also engages them in the exciting process of envisioning and organizing an enjoyable activity, which should positively affect intrinsic motivation to join the training. Exercising problem-solving skills in a new and stimulating context adds an element of curiosity and allows to practice problem solving, including facing unexpected events (Sakaki et al. 2018).

The specific objects of the present study were: (1) to understand the social and cognitive concerns and needs of older adults; (2) to understand their familiarity and difficulties in using new technologies in everyday activities; (3) to explore their expertise in the activity of planning a trip or similar ones; (4) to investigate their motivation and expectations in participating in a web-based training activity to improve cognitive functions, specifically their problem-solving abilities in planning a trip; (5) to obtain suggestions for the development of SWIFT.

## Method

### Participants

A convenience sample of 27 healthy older adults aged 65–84 years (M = 72.96, SD = 5.48; 12 men, 15 women) living in Northern Italy and in the South of the Netherlands was recruited by contacting senior citizen associations. Inclusion criteria were limited to age (> 65 years) and absence of neurological or psychiatric disorders because the training is designed for older people without any specific disorders that would require different features. As shown in Table 1, most of the participants were married (70%), 55% have a diploma or a master degree and 96% use new technologies. Only the participants in Padua focus groups (*N* = 14) were familiar with computerized cognitive enhancement training, as they had previously participated in a cognitive enhancement study. The characteristics of the sample make it selective when compared to the population of the same age in Italy and the Netherlands of which 62.1% and 60.1% respectively are married, 32.5% and 36.4% have a high level of education, and 44.8% and 90.3% use Internet (Eurostat 2023).

**Table 1:** Characteristics of participants

	Italy *N* = 14	Netherlands *N* = 13	Total *N* = 27
*Demographic characteristics*			
Education			
Primary education	1	1	2
Upper secondary education	6	4	10
High School Diploma	2	4	6
Master's degree	5	4	9
Retired			
Yes	14	12	26
No	0	1	1
Marital status			
Married	12	8	20
Divorced	1	0	1
Widowed	1	4	5
Cohabiting	0	1	1
Children			
0	0	1	1
1	9	1	10
2–4	5	11	16
Grandchildren			
0	5	2	7
1	4	2	6
2–4	5	5	10
5–9	0	4	4
Living situation			
Single	1	3	4
Wife/husband	12	10	22
Grandchildren	1	0	1
Use of computer or smart phone			
Yes	14	12	26
No	0	1	1
*Leisure activities*			
Read newspapers			
Yes	12	13	25
No	2	0	2
Read books			
Yes	11	9	20
No	3	4	7
Housework			
Yes	12	13	25
No	2	0	2
Social activities			
Yes	8	8	16
No	6	5	11
Sports/exercise/yoga			
Yes	8	8	16
No	6	5	11
Movies/theater			
Yes	8	7	15
No	6	6	12
Crossword/puzzles			
Yes	7	8	15
No	7	5	12
Volunteer work			
Yes	4	7	11
No	10	6	16
Gardening			
Yes	4	4	8
No	10	9	19
Artistic activities			
Yes	1	1	2
No	13	12	25

### Data collection

Four focus groups, two in Italy (Padua and Bologna) and two in the Netherlands (Tilburg), were conducted. A focus group is a qualitative research technique used to draw upon attitudes, feelings, beliefs, experiences and reactions of small groups of participants about a given problem, experience, service, or other phenomenon keeping into account their particular culture. Focus group allows researchers to explore a phenomenon in a way in which would not be feasible using other methods (for example observation, one-to-one interviewing, or questionnaire surveys) because the exchange of ideas among participants leads to the generation of novel insights that is particularly relevant to develop or improve new products and services. Moreover, focus group is useful to explore the degree of consensus on a given topic and keep into account the specific culture and habits of particular groups (Morgan 2009).

All focus groups were conducted in university classrooms in the departments of the respective cities. The sessions lasted approximately two hours and were conducted by a qualified moderator and a second research assistant who monitored the technical aspects of the session and took notes. Before starting the discussion, the main moderator reminded the participants of the aim of the focus group and that the discussions would be audio and video recorded for later analysis of the results. The discussion for the four focus groups followed a common interview script, as shown in Box 1.

**Box 1:** Interview Guides for Focus Group Discussions

**a. Previous Experience with a cognitive training**
1. Have you ever participated in a training to improve your mental skills? What was the experience like? What did you find useful? What was difficult instead?
2. If you have never participated in such a training, in what everyday situations do you have to plan activities? How do you do it? What solutions do you find useful to carry out the different activities? What difficulties do you encounter?
**b. Problem Solving When Planning a Trip**
1. When you have to go on a trip, who plans it? Do you do it alone or with the help of others? Do you rely on travel agents?
2. Who is responsible for selecting and booking the facilities?
3. What difficulties do you encounter?
4. What possible unexpected events might you encounter during a trip? How do you plan to deal with them?
5. How can you find bus/train/plane schedules?
6. When traveling, where do you prefer to stay (hotel or apartment)?
7. When traveling, do you prefer to have a precise itinerary? (Or live the experience day by day based on the attractions of the city you are visiting?)
**c. Level of familiarity with information technology**
1. Do you use a computer, smart phone, or other similar tools? For what activities?
2. How can they be useful?
3. What difficulties do you encounter when using these devices? What solutions do you find?
**d. Motivation and expectations for training**
1. If you were invited to participate in a training to improve your planning skills, would you like to attend? What about computer-based training? Why? What would you like to plan? What skills would you like to improve?
2. What features (including technical ones, e.g., computer or tablet) should the training have? What tasks might be useful?
3. What do you expect (in terms of results) from such a training?
4. What difficulties do you think you might encounter in using the training?
5. Would you like to do this with others? How might that be? How might it help you and what might be the difficulties instead?

All participants gave written informed consent and completed a brief questionnaire about their demographics, leisure activities, and previous experience of using IT. The Ethics Committees for Psychological Research of Padova and Tilburg University approved the study.

### Data analysis

After each group discussion was transcribed verbatim and translated into English, thematic analysis (Braun and Clarke 2006) was conducted using Atlas.ti 8 software. The researchers first read through the focus group transcriptions to gain an overall impression. In the second reading, the researchers independently identified and categorized central themes. The researchers revised some of the themes in light of the other focus group transcripts, including notes and comments. After comparing similarities and differences among the themes, the researchers refined them to develop a common analytical framework. Details of the analysis process are provided in Box 2.

**Box 2:** Details of the analysis process

1. S.C. and D.S. read the transcripts and familiarized themselves with the data
2. S.C. and D.S. independently identified preliminary codes and themes
3. S.C., D.S., and F.S. compared and discussed the preliminary codes and themes
4. D.S. coded all the material according to the preliminary codes and themes
5. S.C. revised the preliminary codes and themes and compared them with her field notes
6. S.C., D.S., and F.S. discussed the revised codes and themes and agreed on the final codes and themes
7. S.C. and D.S. reviewed the transcripts to challenge the findings
8. All the authors discussed the findings and themes and agreed on the interpretation of the data

Reflexivity was sought through repeated comparison of the themes in relation to the data, and discussions were held between the researchers about alternative interpretations of the findings.

## Results

Thematic analysis showed a great similarity between the results of the focus groups conducted in Italy and the Netherlands, thereby a common codebook was created, which included the codes and finalized the coding categories into themes. Differences between the groups are noted where relevant. Five thematic areas were identified, with the associated themes and codes, as summarized in Table 2. Participants' quotes are presented to illustrate each theme and each participant is identified by a G for the group they belong to (G1 = Padua, G2 = Bologna, G3 = Tilburg 1, G4 = Tilburg 2), a P followed by a number, M for male participants, F for female participants and age.

**Table 2:** Themes and codes

Thematic areas	Themes	Codes
Interests and activities	Personal Characteristics	Interests
		Retirement
		Occupation
		Past health problems
	Leisure Activities	Training
		Sports
		Grandchildren
		Volunteer work
		Social activities
		Domestic activities
		Creative activities
Difficulties and concerns	Concerns	Cognitive health
		Concers about attention
		Concers about memory
		Loneliness
		Concers about retirement
		Concerns about aging
	Cognitive Function	Present functionality
		Past functionality
	Cognitive Difficulties	Memory
		Attention
		Cognitive difficulties about aging
Experiences and motivation for training	Needs	Staying active
		Digital empowerment
		Memory improvement
		Attention improvement
		Socialization
		Support
		Relationships with younger generations
	Previous Experience with a cognitive training	Motivation for participating in the training
		Self-assessment of performance
		Cognitive experience
		Emotional experience
Expertise and resources	Technological Skills	Phone
		Social network
		Difficulty
		During covid
		Thirty years ago and today
		IT for travel arrangements
		IT for travel organization difficulties
	Travel Experience	Past travel experiences
		Needs to afford traveling
		Difficulties linked to traveling
		Unexpected events
		Concerns about traveling
	Travel Planning Skills	Travel planning experience
		Travel organization difficulties
		Arranging future travels
	Organized group travel	Advantages
		Disadvantages
Suggestions for the design of the new training	Future training	Expectations
		Required knowledge
		Suggestions
		Availabilities
	Collective training sessions	Evaluation of collective training
		Suggestions

### Interests and activities

Almost all of the participants (26/27) are retired, and in their free time they do many activities to stay active: long-standing interests or activities that they were previously unable to devote time to.

Examples include sports, reading, volunteering, traveling, and taking care of grandchildren: "I do some sports" (G2, P1, M, 66 years); "I also take care of the grandchildren, in addition to other activities such as reading; I have been traveling since I retired and have no other priorities for the time being" (G1, P7, F, 73 years); "We do a lot of hiking in the mountains, including multi-week treks, and cycling" (G4, P3, M, 78 years); "I now volunteer at the nature museum because nature is a big interest of mine. I also like to walk, even long distances. I also volunteer at the women's centre where I do organizational, administrative and secretarial work" (G4, P1, F, 71 years).

Two participants reported using their free time to further their education: "Recently, I have taken several courses (at the college for seniors): history of art, science applied to modern technologies, writing courses. Courses that I did not have time for before" (G2, P2, M, 77 years); "In recent years, I have started to study religion, philosophy, psychology. After retirement, I mainly do activities that interest me" (G4, P4, M, 72 years).

In spite of their retirement, three participants reported that they find it difficult to give up their former professional activity and therefore continue to do it voluntarily: "Professionally, I was a manager and an entrepreneur. Basically, you can't say I'm retired or not retired, I'm always there, at different paces, for consultations and requests" (G1, P1, M, 80 years); "I still have a full schedule, because I'm still a good mechanic, and I'm often asked for help" (G1, P1, M, 66 years); "I retired at 65, now I'm 77, and for eight years after I retired I had various supervisory positions, in youth welfare, and that taught me a lot" (G4, P2, F, 77 years).

### Difficulties and concerns

This topic area focuses on participants' concerns. Participants reported a high level of concern about the risk of cognitive decline, believing that efficiency in performing activities of daily living is consistently dependent on cognition.

Participants often reported the differences between their current and past cognitive functioning: "Now that my attentional resources are much diminished, I have more difficulties" (G1, P5, F, 70 years); "Compared to before, my memory has really deteriorated" (G4, P2, F, 77 years); "Even my attention is not as good anymore" (G4, P1, F, 71 years).

These cognitive difficulties, which participants often identified as memory or attention problems, were expressed in a variety of ways, from the inability to remember a name or face to forgetting important things or being unable to concentrate on a particular task. Several participants (11/27) accurately reported individual episodes in which they experienced cognitive difficulties. For example, "I go to a doctor's appointment and I can never remember the name of the medicine prescribed, and I am a doctor" (G1, P7, F, 73 years); "I was supposed to issue receipts for membership dues and when I prepared a receipt for one person, I got the name wrong; I wrote down the name of a person I know but who has nothing to do with the association" (G1, P5, F, 70 years).

One participant emphasized the negative effects of the COVID-19 pandemic on her own cognitive health: "The whole period of the pandemic was tragic for me, I feel that both my physical and intellectual abilities decreased. It was a strong alarm, an awareness that the decline is there and it evolves a lot when you let go" (G1, P5, F, 70 years).

For some participants (5/27), these cognitive difficulties can become a real nightmare, an obsession: "I spend a lot of time during the day trying to remember where I put things" (G1, P8-F, 69 years); "For me, these forgetfulnesses drive me crazy after a few minutes" (G1, P2-F, 70 years); "I have a feeling of deterioration" (G4, P2, F, 77 years).

Moreover, not everyone is able to cope with these difficulties. Many participants feel frustrated and helpless in the face of their forgetfulness, often resigning themselves to the idea that "memory is inversely proportional to age" (G1, P1, M, 80 years). Two participants reported that they gave up doing things that were important to them for fear of not being able to do them anymore, e.g. "I said to myself, you have a rusty memory, you can't remember well, you forget. So I said to myself, "No! Forget it. I didn't do it" (G2, P6, F, 69 years); "I wanted to do it, but I didn't feel like enrolling because I'm too old to study for another 4 years" (G4, P4, M, 72 years).

Another element of concern, explicitly reported only by the Italian participants but common to all the participants, is loneliness. The idea of loneliness takes on different connotations: some link it to the concept of autonomy, in fact they are afraid of being alone because on their own they might not be able to carry out all the essential activities of daily life; others experience it more as social isolation, loss of contact and relationship with others: "My fear is that of loneliness, I don't like it, it doesn't belong to me, being alone is the worst thing that can happen to me" (G2, P6, 69 years); "I am afraid of being alone" (G2, P5, 65 years).

Concerns about loneliness turned out to be related to concerns about retirement. Retirement is an event that, in the eyes of the participants, increases or severely sanctions their loneliness and social isolation. Leaving the work dimension deprives them of the possibility of establishing daily interpersonal relationships as easily as before: "What does it mean to have retired and to have suffered from retirement? It changes the role and the consideration that other people have for you, especially if you had a job like mine with constant contact with people" (G2, P6, 69 years). "If you retire and have no obligations, and no one is looking for you or expecting anything from you, then, as happened to a friend of mine, you will die within 6 months" (G4, P2, F, 77 years).

Two participants reported feelings of anxiety due to retirement: "Since I've retired, I've taken on so many commitments, and now I can't keep them. I take them on because I have the anxiety of wanting to be there, wanting to do something" (G1, P5, F, 70 years); "While I was partially satisfied at work, now in the days that are almost half empty, I sometimes get anxious" (G2, P3, M, 78 years).

### Experiences and motivations for training

When participants were asked what skills they wanted to improve, they identified several skills as well as a need for support and socialization. The first need was to improve their cognitive skills, especially attention and memory: "I need to practice and improve my memory" (G1, P3, M, 76 years); "I would like to train my attention" (G4, P2, F, 77 years). The second need is to improve their digital skills. Almost all participants considered these skills to be crucial for being informed and actively participating in social life: "You have to keep up with technology" (G2, P6, F, 69 years).

These two needs turned out to be linked. Acquiring IT skills becomes a way for many of the participants to prove to themselves that they are still able to learn and perform adequately: "I also need to challenge myself digitally, it is important to learn how to use new devices" (G1, P7, F, 73 years).

For eight participants the need to stay active and fill their daily agenda increased with age: "I realized that you cannot give up, you have to be active, very attentive and do everything you can. It is important to be aware that we are really the creators of our own well-being, much more than when you are young, because when you are young you don't need it, but at this age it is important" (G1, P5, F, 70 years); "What I also think is important is to go to the theatre, to participate in cultural, sports and community activities" (G1, P7, F, 73 years).

Participants also expressed the need of socialization: "I need to socialize, I need to be with others. It is very important for me" (G2, P4, F, 65 years); "We need to talk, to share our experiences with others" (G2, P5, F, 65 years). Technological support, or support in general, becomes a compelling need because it also satisfies socialization needs: "With the lockdown, we realized that if you don't use technological tools, you are isolated and marginalized. We have problems with loneliness and marginalization, and I can imagine that if you don't have some technological skills, you will be more and more excluded and isolated" (G2, P3, M, 78 years); "At this point, it is worthwhile to develop technology for our world, the world of the elderly" (G2, P3, M, 78 years).

The identified needs fuel the participants' motivation to engage in cognitive enhancement and training experiences (digital and otherwise). Participants in the two groups conducted in Italy participated in various cognitive enhancement and digital training initiatives proposed by psychology departments or third age associations in the area. The motivation of the participants who took part in these initiatives was based on the desire to improve themselves and to learn: "I expected to understand how the mind works and what to do to make it work better" (G1, P1, M, 80 years); "I hope that the training I participated in can improve my memory" (G1, P4, M, 67 years). The trainings proposed by the departments seem to respond adequately to the need for empowerment and the need to remain active expressed by the participants.

### Expertise and resources

All participants perceived IT as an important tool for communication, staying up to date and keeping in touch with the new generations. For four participants, the use of technological devices became an essential way to maintain social contacts during the pandemic. Six participants highlighted the benefits of technological progress in different areas of life: "The first numerically controlled machine led my company to a considerable development. In ten years, 15 people were hired and a new hall was built" (G2, P6, F, 69 years); "IT has changed everything, today everything is possible" (G2, P3, M, 78 years). For some, technological literacy is necessary to be an active part of today's society: "We should have at least a minimum of knowledge to use IT" (G1, P5, F, 70 years). However, this positive view is accompanied by concerns about their ability to adapt to modern technologies: "We were not digital natives. We have difficulties in approaching IT" (G1, P6, F, 67 years).

Twenty-four participants said that they are used to using IT and that they also use it to establish and maintain social relationships, while the remaining three reported having difficulties in approaching new technologies. Those who use technological devices report using them for various activities: research in the Google encyclopedia, Excel calculations, reading and writing e-mail messages, online shopping (Amazon, drugs), engaging in social networking activities (Facebook, WhatsApp), using various mobile phone applications (diary, e-mail, ATM payments, Spotify), organizing travel: "Today, there are IT tools that show route maps wherever you are. It is very helpful to be able to use these tools" (G1, P2, M, 70 years); "If I have to book a trip, I prefer to use the computer" (G1, P7, F, 73 years); "My annual subscription to Spotify has allowed me to listen to information that interests me" (G3, P5, F, 76 years).

Participants who are already familiar with IT often ask for help when they encounter difficulties. Help usually comes from children or grandchildren: "We have children and grandchildren we can ask" (G3, P3, M, 84 years); "Sometimes the computer crashes. I wait for my son to come and ask him to fix it. It is the same with the telephone: if there is a problem, I ask my son for help" (G3, P7, F, 65 years).

The participants are very interested in traveling and talk about their different experiences (e.g., trekking, hotel, caravan) in different countries (e.g. Italy, Spain, France, Scotland, Santiago, United States, China): "As a boy I travelled a lot, I travelled the world with the map. The first trip I made was when I was only 15 years old, with a friend of mine we went on a motorcycle from Capri to Naples. Then we decided to go to Paris. We had 230,000 lire. We ate sandwiches for ten days, but we made it to Paris (G2, P3, M, 78 years old). However, with age, insecurity increases and some participants (10/27) feel less autonomous and independent. "I don't travel anymore, I'm too old and I can't walk anymore" (G3, P5, F, 76 years); “Children are not always willing to help, so I prefer to rely on organized trips. I do not want to always depend on someone else. I would like to go on a trip by myself, but I don't have the courage” (G1, P6, F, 67 years).

Fear of traveling is related to concerns about aging, self-perceived limited competence in using technological tools, and linguistic fluency in foreign languages: "If you travel alone, another problem may be language skills. It is a problem for me. I can get by in English, but only at a very basic level. Organizing everything, talking to people, understanding what they are saying, I find all these aspects quite difficult" (G1, P8, F, 69 years); "Nowadays, if I had to go on a long trip alone, I would feel embarrassed, although there are several useful tools, such as GPS" (G2, P3, M, 78 years).

Almost all participants feel the difference between their former travel independence and their current travel skills. Participants often chose to travel with agencies: travel organization, solutions to obstacles and difficulties are no longer their responsibility: "Now I prefer organized trips because I feel calmer and more relaxed" (G2, P3, M, 78 years); "I prefer organized trips because there is always someone who can support you" (G1, P3, M, 76 years); "An organized group gives you more confidence also in case of communication problems abroad" (G1, P8, F, 69 years).

Almost all participants reported that they had experienced a decline in their self-confidence and autonomy as they got older; many of them did not feel able to move independently in unfamiliar places, and many preferred not to travel outside their familiar surroundings.

Others (10/27) expressed a need for independence and freedom and have little tolerance for the organization of travel agencies: "Next week I will travel with an organized group. When I read the final program, I realized that it did not meet my expectations. This is an example of the limitations of an organized trip. (G1, P2, M, 70 years); "Often, when I travel with an organized group, I have the desire to leave the group" (G2, P2, M, 77 years); "Timing and goals are not always accepted and shared by everyone" (G2, P2, M, 77 years).

All participants believe that autonomy and safety are the most important issues when traveling. Few participants organize their trips themselves using computer devices or apps: "If the trip is simple enough, I can manage it. I know how to get around the city, I know the facilities, I'm also able to make reservations by phone" (G1, P8, F, 69 years); "On my own, I organized 5 stops in the most beautiful places in central Italy" (G1, P1, M, 80 years).

### Suggestions for the design of the new training

Most of the participants showed interest in the project of developing a web-based cognitive enhancement training focused on planning and problem-solving skills and they expressed their willingness to participate. All showed a strong interest in completing activities related to travel planning.

Some participants (7/27) made suggestions about possible activities to include in the training such as “different types of trips (biking, hiking, camping)" (G1, P2, M, 70 years) or "Deciding what to go and see" (G2, P1, M, 66 years). They suggested to pose attention to different aspects: "Budget is one of the most important things. The goals, type and number of places to visit should be carefully evaluated, taking into account the budget. "Eating is the most important thing" (G2, P3-M, 78 years). "Consider not only when to go, but also the best time to go. For example: I plan a trip because I want to see the Northern Lights. If I go in the wrong month, I won't be able to see them. If I leave in the evening, I will not be able to visit certain places" (G2, P4, F, 65 years). "It is also important to make a daily plan. Understand what there is to visit in a place, how not to waste time, how to make reservations" (G1, P7, F, 73 years).

Regarding the expectations and the way the training can be carried out, one participant said: "The results also depend on the frequency. If you do it only once a month, it fades away immediately. If you want to train something, you have to do it often enough. Especially with our memory problems, I would like to start training right away" (G4, P2, F, 77 years old). The same participant added: "Of course you also have to think about what you want to achieve and at some point you have to check what you have really achieved" (G4, P2-F, 77 years).

All participants emphasized only positive aspects of a collective training sessions: "Everyone brings their own difficulties, what they don't know how to do" (G1, P7, F, 73 years); "I like to compare myself with people who know more and have something to say. I would feel fulfilled" (G1, P6, F, 67 years).

## Discussion

This study explored older adults' concerns and difficulties in performing activities of daily living, their range of needs, their abilities and difficulties in using technology, and their motivations and expectations for participating in a web-based training activity to improve problem-solving skills.

An important initial finding is that participants reported numerous concerns and self-reported cognitive difficulties but also a strong desire to improve their skills. They showed motivation to stay "mentally active" and to learn new things (see van Kampen et al. 2023 for similar findings). All participants volunteered for the study and, in the case of the Italian sample, also participated in cognitive enhancement initiatives. All are interested in using new technologies (see Heinz et al. 2013; Mitzner et al. 2010, for similar findings), but they encounter numerous problems (e.g., excessive complexity and lack of clarity) when using them (see also Vaportzis et al. 2017; Roberts et al. 2019). These difficulties lead to feelings of inadequacy and frustration, and interfere with learning and subsequent IT use. When designing web-based cognitive enhancement programs, these feelings of inadequacy and difficulties with IT use must be considered.

Participants emphasized the need for support and assistance in learning new activities. Therefore, IT based interventions designed for older people should include articulated help systems in the training to enable effective learning. This could be an online help system to provide the necessary information to solve doubts and difficulties. It is important to provide all the necessary information both to support motivation to learn and to enable the development of a sense of self-efficacy. Indeed, as reported in Huang and Oteng's (2023) review, older adults' self-efficacy and anxiety represent significant barriers to technology adoption.

Participants reported that they rely on the family network in case of IT difficulties, but it also became clear how important it is to acquire the necessary skills independently. This can overcome the barriers that older people face in participating in web-based cognitive training.

New technologies should support and facilitate IT learning itself by ensuring that people do not give up learning because they feel inadequate. The ability to learn independently and flexibly to gain confidence in using technology and in one's own abilities through practice is essential. In fact, direct experience with computers creates more positive attitudes towards technology in older adults (Czaja and Sharit 1998; Lee et al. 2019). Our study also emphasized that the cognitive trainings should be based on the interests of the participants. Older adults prefer to challenge themselves in activities that are related to their desire for resilience and autonomy, as well as a sense of doing well (De Angeli et al. 2020). The trainings should be challenging and the level of difficulty should be adjusted to suit performance. It is also important to provide emotional feedback during and at the end of the training sessions in order to maintain motivation, strengthen and increase self-esteem (Kimbler et al. 2012; Ferdinand and Hilz 2020). The emotional feedback indicated concern, trust, empathy, and caring.

To fulfil the requirements pointed out by the participants of this study, SWIFT will be based on the activity of planning a trip, according to the ideas and wishes that older adults expressed in the focus groups. Users will have to plan a two-day vacation in a European city: they will organize virtual train and hotel reservations, and they will carry out various activities (e.g., visit certain places, attend certain events). To accomplish these tasks, the users have to navigate on a map where the objectives are those typical of planning a trip in real life (e.g., budget management, reservations, bus schedules, opening hours of some specific places).

To meet the need for socialization expressed by the participants, it is important to design tools that promote social interactions. The design of collective training sessions would allow the participant to benefit from the collaboration and support of other people online, through discussion to solve the problem, and thus reduce feelings of loneliness. SWIFT will be structured to encourage social interactions among participants through online collaborative training sessions, taking advantage of the features of social networks. These, as shown by studies in the literature (Wolfe et al. 2023), can have a positive impact on cognitive function in addition to reducing feelings of loneliness.

The limitations of the present study include the small sample size. Additionally, only the Italian participants had previous experiences with cognitive training, thus creating a difference between the groups of the two countries that cannot be referred to cultural features. No other particular difference was found referring to the two countries. This might be due to the common European background and to participants’ similarities that made the sample selective. This is a limitation of convenience sampling, which might not be representative of the population but is coherent with the aim of qualitative research that is not to generalize but rather to provide a rich, contextualized understanding of some aspect of human experience through the intensive study of particular cases (Polit and Beck 2010). Further studies should explore needs and motivation in participating in a similar training in other cultural contexts and with disadvantaged groups who might have a different travel experience.

In conclusion, our results suggest that older adults are increasingly interested in managing their health and have expectations for active aging. In fact, most of our participants were eager to adopt new technologies and willing to participate in computerized cognitive enhancement activities based on travel planning, recognizing their potential benefits in maintaining a high quality of life and preserving or even improving their cognitive abilities. However, this positive attitude might also be affected by the selective sample. Engaging older adults in challenging but enjoyable activities such as planning a trip may be a way to extend the interest to keep themselves active and also motivate other older adults (e.g. less active, with lower educational and/or socio-economic level) to participate in cognitive trainings. Yet, attention needs to be paid when creating the program that content of the training (e.g., budget for planning a trip) is realistic and relevant to all older adults, irrespective of for instance their socioeconomic background and mobility status. The attempt to motivate older adults of any backgrounds to participate in cognitive trainings needs to be further supported by society through public policies, programs, and resources aimed at promoting active aging.

The involvement of the final users from the initial stage of the design process to the successive, development and testing phases is fundamental to make training resources more usable, appealing and closer to the users’ interests, increasing participation or usage. The participatory design of a collective web-based training tool of problem-solving skills will be a first step in this direction.
